# Graphene Oxide–Hydroxyapatite Nanocomposite Coatings and Extracellular Matrix Protein Interactions for Enhanced Osseointegration in Dental Implants

**DOI:** 10.1111/jcmm.71352

**Published:** 2026-09-03

**Authors:** Ravinder S. Saini, Rayan Ibrahim H. Binduhayyim, Doni Dermawan, Masroor Ahmed Kanji, Syed Altafuddin Quadri, Artak Heboyan

**Affiliations:** ^1^ Department of Allied Dental Health Sciences COAMS King Khalid University Abha Saudi Arabia; ^2^ Applied Biotechnology, Faculty of Chemistry Warsaw University of Technology Warsaw Poland; ^3^ Department of Dental Technology, COAMS King Khalid University Abha Saudi Arabia; ^4^ Department of Research Analytics, Saveetha Dental College and Hospitals, Saveetha Institute of Medical and Technical Sciences Saveetha University Chennai India; ^5^ Department of Prosthodontics, Faculty of Stomatology Yerevan State Medical University after Mkhitar Heratsi Yerevan Armenia; ^6^ Department of Prosthodontics, School of Dentistry Tehran University of Medical Sciences Tehran Iran

**Keywords:** dental implants, extracellular matrix proteins, graphene oxide, hydroxyapatite, nanocomposite, osseointegration

## Abstract

Graphene oxide (GO) has been recognised to have a strong protein‐binding ability attributed to its high surface area and oxygen functional groups (OFGs) but is plagued with dose‐dependent cytotoxicity. In contrast, hydroxyapatite (HA) is highly biocompatible but has shown relatively weak protein–protein interactions on the molecular scale. The strategic merging of GO and HA into the GO–HA nanocomposite has the potential to synergistically and precisely facilitate bioactivity and biocompatibility across the implant–tissue interface. Molecular docking, pharmacophore modelling, and molecular dynamics simulations were used to explore the molecular interactions of GO, HA, and GO–HA nanocomposites with ECM proteins, including fibronectin (FN), collagen (COL), laminin (LAM), periostin (POSTN), vitronectin (VN), and osseointegration‐related integrin receptors under physiological conditions. The GO–HA nanocomposites exhibited an enhanced number of hydrogen bonds and decreased residue‐level fluctuations relative to GO or HA individually, indicating improved interfacial interaction stability at the protein–surface interface. Molecular dynamics simulation results indicated that ECM proteins in the presence of GO–HA showed better conformational stability, which might lead to favourable integrin‐mediated adhesion. Preliminary in silico toxicity‐related screening of the individual GO and HA model structures suggested differential safety alert patterns, with GO showing more cautionary alerts than HA. These outputs were interpreted qualitatively because the predictive tools used were primarily designed for small molecules rather than nanomaterials. Overall, these in silico results at the molecular scale revealed insights into the surface–biomolecule interactions of GO–HA, which may pertain to early events in osseointegration and can be used to create awareness for downstream in vitro and in vivo validation of dental implant coatings.

**Abbreviations**
ADMET: absorption, distribution, metabolism, excretion, and toxicity
BSP: bone sialoprotein
CASTp: computed atlas of surface topography of proteins
CNS: central nervous system
ECM: extracellular matrix
GAFF2: general amber force field
GO: graphene oxide
HA: hydroxyapatite
HADDOCK: high ambiguity driven protein–protein docking
MD: molecular dynamics
NIS: non‐interacting surface areas
NPT: number of particles, pressure, and temperature
NVT: number of particles, volume, and temperature
PDB: protein data bank
PME: particle mesh Ewald
RMSD: root mean square deviation
RMSF: root mean square fluctuation
RoG: radius of gyration
SLRP: small leucine‐rich proteoglycan
TGF‐β: transforming growth factor‐beta
VDW: van der Waals

## 1 Introduction

Dental implants have gained high acceptance in restorative dentistry as a predictable long‐term tooth replacement therapy [1]. Successful osseointegration, or direct structural and functional connection between the implant and surrounding bone tissue, is critical for the long‐term success of dental implants [2]. Despite the good mechanical properties and corrosion resistance of titanium implants, the naturally bioinert surface of these materials can limit direct biological interactions with bone tissue, potentially leading to insufficient early bone–implant integration and implant failure [3, 4]. A wide variety of surface modification techniques have been developed and tested to overcome the limitations of titanium implants and improve their bioactivity. In this context, hydroxyapatite (HA) coatings have gained clinical interest because of their chemical resemblance to native bone minerals and ability to promote osteoconduction [5, 6, 7]. Several studies have highlighted their practical applications in dentistry and orthopaedics, including plasma‐sprayed HA coatings for dental implants, which show enhanced bone–implant contact [8, 9], sol–gel‐derived HA coatings that improve bonding strength and bioactivity [10], and electrophoretic deposition of HA for uniform, adherent layers on metallic implants [11]. However, the biological and mechanical performance of these HA coatings remains highly variable and often depends on the deposition method, crystallinity, coating thickness, and surface morphology [12]. Plasma‐sprayed HA coatings are widely used for this purpose but have several well‐known limitations, including poor long‐term mechanical stability, tendency for delamination, and unsatisfactory protein adsorption efficiency [13, 14]. Several studies have demonstrated the rapid resorption of amorphous or poorly crystalline HA layers and their suboptimal protein binding compared to crystalline or surface‐modified coatings [15, 16]. The combined limitations of current dental implant technologies demonstrate a clear requirement for advanced composite coating methods that enhance the mechanical reliability and molecular bioactivity at the implant–bone interface.

Therefore, recent efforts have been directed toward the optimisation of HA‐based coatings using alternative fabrication routes, including pulsed laser deposition, biomimetic coating, and electrophoretic deposition, which allow better control of the crystallinity and adhesion strength relative to plasma spraying [17, 18]. Composite coatings with the incorporation of a secondary phase have also been widely investigated to improve their mechanical stability and biological performance. Such materials include HA–titania, HA–zirconia, and HA reinforced with nanomaterials such as carbon nanotubes, bioactive glass, and graphene derivatives [19, 20, 21]. These composite scaffolds not only improve the toughness of the materials but also positively affect protein adsorption and osteogenic differentiation. In addition to promoting osteoconduction, several other functionalisation strategies have been developed to actively tune the biological response at the implant–tissue interface. For example, the immobilisation of peptides containing Arg‐Gly‐Asp (RGD) motifs, incorporation of bone morphogenetic proteins (BMPs), and loading with antibacterial agents or silver nanoparticles have been utilised to encourage rapid osseointegration and mitigate peri‐implant infections [22, 23, 24].

Graphene oxide (GO), a chemically functionalised derivative of graphene, is an emerging nanomaterial of increasing interest as a bioactive additive for HA‐based implant coatings [25]. GO has distinctive characteristics, including exceptional mechanical strength, high surface area, and abundant oxygen‐containing functional groups [26, 27]. The beneficial properties of GO make it an excellent additive for improving HA coating bioactivity. Previous studies have shown that incorporating GO into HA coatings enhances protein adsorption, osteogenic differentiation, and the mechanical performance of the resulting composite systems [28, 29, 30]. The early stages of osseointegration require critical interactions between implant surfaces and extracellular matrix (ECM) proteins [31]. Fibronectin, collagen, and laminin ECM proteins play critical roles in cell adhesion and the regulation of cell proliferation and differentiation [32, 33]. When proteins attach to implant surfaces, their structural alignment and positioning significantly affect cellular behaviour. Integrin‐dependent cell adhesion requires the exposure of the Arg‐Gly‐Asp (RGD) sequence in fibronectin [34]. The negatively charged surface of GO has been reported to induce extended conformations of fibronectin, potentially enhancing RGD domain exposure and integrin‐mediated cell attachment [35]. Integrins, which serve as transmembrane receptors that enable cell–extracellular matrix binding, play a fundamental role in osseointegration [36]. The integrin receptors α5β1 and αvβ3 function as specific recognition sites that bind ECM proteins and trigger signalling pathways that control cell survival, proliferation, and differentiation [37]. Research on GO–HA composites has gained momentum, but the molecular interactions between GO–HA coatings, ECM proteins, and integrin receptors need further exploration to enable the development of optimally designed implant surfaces.

Computational modelling approaches have also been adopted to explore the molecular‐level interactions between implant coatings, ECM proteins, and cell receptors with high spatiotemporal resolution in recent years [38, 39]. Extensive application of molecular docking and MD simulations has helped to characterise the adsorption of ECM proteins on the biomaterial surface and understand how the surface chemistry, charge distribution, and nanoscale morphology of the material modulate protein conformation and binding affinity [40, 41]. However, many previous computational studies were limited to short timescales, simplified surface models, or a single protein–surface interaction and may not fully represent the complexity of the implant–tissue interface. Accordingly, an integrative computational framework that can systematically assess protein adsorption, adhesion domain accessibility, and integrin‐mediated binding on advanced composite coatings, such as GO–HA, is desired.

The objective of this research was to provide a detailed understanding at the molecular level of the events that GO–HA coating induces in protein adsorption and integrin–ECM engagement, which are important in osseointegration processes. Molecular docking and molecular dynamics simulations were used to examine fibronectin and other ECM proteins, such as collagen and laminin, on the GO–HA surface and to identify conformational changes in adsorbed proteins that modulate binding affinities. The exposure of integrin‐binding sites in adsorbed ECM proteins was evaluated to determine whether the presence of GO enhances recognition and binding by integrin receptors. Interactions between ECM proteins and integrins, such as α5β1 and αvβ3, were also modelled to identify potential signalling pathways that may promote osteogenic differentiation. A comparative study of the GO–HA composite and pristine HA was performed to identify the specific bioactive role of GO in the composite coating.

## 2 Methodology

The representative ECM proteins in this study were chosen based on their well‐documented involvement in cell adhesion, bone regeneration, and implant osseointegration. The 3D structures of selected ECM proteins and integrin receptors were retrieved from the Protein Data Bank and energy‐minimised, whereas a graphene oxide–hydroxyapatite (GO–HA) nanocomposite model was constructed to represent the physicochemical features relevant to implant coatings. Molecular docking simulations were performed to assess the binding orientations, interaction profiles, and relative binding affinities between ECM proteins, integrin receptors, and the GO, HA, and GO–HA systems. The docked complexes were subjected to molecular dynamics (MD) simulations to investigate their conformational stability, residue‐level flexibility, and adhesion domain accessibility under physiological conditions. Pure hydroxyapatite was included for comparative purposes to elucidate the specific contributions of GO to protein binding and stability.

### 2.1 Selection of Extracellular Matrix (ECM) and Integrin Receptors

To identify the molecular mechanisms responsible for promoting osseointegration by GO–HA nanocomposite coatings, biologically relevant ECM proteins and integrin receptors were selected systematically. The selected ECM proteins, such as fibronectin, collagen, laminin, periostin, vitronectin, osteocalcin, osteonectin, and tenascin‐C, were selected for their known roles in mediating osteoblast attachment, proliferation, and differentiation at implant surfaces [42, 43]. Integrin receptors α5β1 and αvβ3 were selected for their critical roles in sensing ECM‐derived RGD sequences and activating cellular signalling pathways, leading to bone formation [44, 45]. ECM proteins and integrin receptors were selected based on their established roles in cell adhesion, signalling, bone remodelling, and early osseointegration at the implant–tissue interface. The experiments were assigned the highest priority for proteins and receptors that have been widely studied in bone biology and dental implant research to support the biological and clinical significance of the computational findings. The three‐dimensional crystal structures of all selected ECM proteins and integrin receptors were downloaded from the Protein Data Bank (PDB) for structural accuracy in docking and molecular dynamics simulations [46]. Advanced computational tools, such as CASTp 3.0 [47] and CASTpFold [48], were used to accurately define the active or binding sites. The carefully curated selection of ECM proteins and integrin receptors provided a solid foundation for understanding the molecular‐level interactions with the GO–HA nanocomposite and uncovering the mechanisms relevant to enhanced osseointegration.

### 2.2 Molecular Representation and Energy Minimisation of Graphene Oxide (GO) and Hydroxyapatite (HA)

Three‐dimensional molecular representations of graphene oxide (GO) and hydroxyapatite (HA) were constructed to capture the principal chemical features relevant to protein–surface interactions at the bone–implant interface. Chem3D molecular modelling software (PerkinElmer Inc.) was used to generate and optimise the molecular geometries, followed by MM2 force‐field energy minimisation using ChemDraw Professional 20.1.1 (PerkinElmer Inc.) to reduce local steric strain and obtain geometrically relaxed structures [49, 50]. In this study, GO and HA were used as component‐level interfacial representations of the combined GO–HA coating system. The purpose of this representation was to enable comparative analysis of biomolecule–surface interactions in the GO, HA, and GO–HA systems. The model was not intended to reproduce the complete bulk structure, crystallographic architecture, coating morphology, or nanoscale heterogeneity of a fabricated GO–HA composite.

Importantly, the computational representation was designed to investigate interactions between the combined material surface and ECM proteins or integrin receptors, rather than to establish a specific covalent bonding mechanism between GO and HA. Therefore, the present model should not be interpreted as direct structural validation of a chemically grafted GO–HA interface or as a quantitative determination of the intrinsic GO–HA interfacial binding energy. MM2 minimisation was employed only to obtain relaxed molecular geometries for subsequent comparative docking and molecular dynamics calculations. Its application to the present GO and HA representations was therefore restricted to structural preparation and should not be interpreted as a prediction of bulk material properties, crystallinity, or coating formation [51]. Accordingly, the GO–HA model represents a simplified molecular‐scale surface assembly intended for comparative protein–surface interaction analysis rather than a complete atomistic reconstruction of the experimentally fabricated nanocomposite.

### 2.3 Molecular Docking Simulation

Molecular docking simulations of ECM proteins and integrin receptors against GO, HA, and GO–HA models were done on the High Ambiguity Driven Docking (HADDOCK) web server platform [52]. Molecular docking was performed using the HADDOCK web server to predict preferred binding orientations. GO and HA were treated as rigid surfaces, and an exploratory docking approach was employed to identify broad regions of favourable protein‐surface interactions rather than targeting predefined binding pockets. Molecular docking is a computational technique that allows the prediction of the preferred orientations and relative binding strengths of interacting molecular entities. In this study, the method was used to predict the interactions between ECM proteins and integrin receptors with the modelled implant coating systems [53, 54], that is, ECM proteins and the GO‐HA complex, thus providing an estimate of their biocompatibility. For the GO–HA system, docking was interpreted as an assessment of biomolecule interaction with the combined material surface representation. The docking calculations were not used to establish the intrinsic bonding mechanism between GO and HA or to validate the bulk structural architecture of the nanocomposite. HADDOCK cluster populations, HADDOCK scores, van der Waals and electrostatic contributions, and buried surface areas were therefore used to compare the relative biomolecule–surface interaction patterns among GO, HA, and GO–HA. HADDOCK was used because it allows flexible, information‐driven docking and is widely used to model protein–protein and protein‐complex molecular surface interactions, including those based on nanomaterials [55, 56]. The analysis of docking solutions considered cluster population numbers and HADDOCK scores to determine the best binding modes from the largest cluster, which had the lowest HADDOCK score, indicating favourable energetic interactions [57, 58]. To complement the analyses, the binding free energy of each complex (ΔG, kcal/mol) was estimated using the PRODIGY web server [59], which provided a thermodynamic evaluation of protein‐GO‐HA interaction stability and affinity. Docking simulations provided a basis for the comparative analysis of binding orientations and interaction regions and the identification of potential protein–surface interaction hotspots for MD simulations by introducing a certain degree of limited molecular flexibility in the model. Subsequent molecular dynamics simulations were performed in the initial stage. This study provides data for further analysis of atomic interactions between ECM proteins and the GO‐HA complex, leading to a mechanistic understanding of early osseointegration events.

### 2.4 3D Pharmacophore Modelling to Assess Active Functional Groups of Graphene Oxide (GO) and Hydroxyapatite (HA)

Pharmacophore modelling has been widely used to describe and spatially map chemical features and specific chemical groups related to molecular recognition (hydrogen bond donors, hydrogen bond acceptors, and hydrophobic) that may guide protein–surface interactions [38, 60]. The goal was to map and identify chemical interaction features at the biointerface that could be driving factors for protein adsorption and integrin engagement relevant to early osseointegration. Using the Ligand Scout 4.5 software platform [61], an advanced pharmacophore modelling tool, the characteristic functional moieties of GO (hydroxyl, carboxyl, and epoxy) and HA (phosphate and calcium‐binding sites) were systematically mapped and characterised, revealing which functional groups within the GO‐HA complex align with the binding features of ECM proteins and integrin receptors. The pharmacophore analysis was therefore used to identify protein‐facing interaction features associated with the GO and HA components of the combined surface representation. Hydrogen‐bond donors, hydrogen‐bond acceptors, hydrophobic features, and related interaction descriptors were mapped to identify chemical functionalities that may contribute to biomolecule adsorption and recognition. Because pharmacophore mapping describes interaction features of the selected biomolecule–surface complexes, it was not used to establish direct GO–HA bonding or to quantify the intrinsic GO–HA interfacial stabilisation. In particular, features assigned to GO or HA should be interpreted as chemical characteristics available at the biomolecule‐facing interface rather than as evidence for a specific chemical linkage between the two nanomaterial components.

### 2.5 Molecular Dynamics (MD) Simulation

Molecular dynamics (MD) simulations were performed to study the temporal stability and interaction patterns of the constructed protein/integrin complex–GO, HA, and GO–HA assemblies under simulated physiological conditions. MD simulations provided critical insights into the behaviour of nanocomposite–protein complexes over time by illustrating conformational flexibility and binding stability while ensuring sustained interaction under simulated physiological conditions. MD simulations were performed using the GROMACS 2023.3 [62] software. The GO–HA nanocomposite was parameterised using the General Amber Force Field (GAFF2) [63], whereas the AM1‐BCC method [64] was used to calculate the atomic partial charges, which provided an efficient trade‐off between accuracy and computational efficiency. A dodecahedron‐shaped simulation box was created with a minimum distance of 2.0 nm from the complex to the box edge to ensure sufficient solvation. Periodic boundary conditions were set in all spatial directions. The AMBER99SB force field [65] was used to parameterise the protein components. The system was solvated using the SPC water model and then neutralised with appropriate counterions. The system truncated short‐range van der Waals and Coulombic interactions at 2.0 nm, and long‐range electrostatics were computed using the Particle Mesh Ewald (PME) method [66]. The MD simulation workflow included several stages: the steepest descent algorithm was used to minimise the energy until the maximum forces of the system fell below 1000 kJ/mol/nm. The system was equilibrated with positional restraints on heavy atoms through the 1000 ps NVT phase and a subsequent 1000 ps NPT phase. Temperature during equilibration was controlled at 310 K using the Berendsen thermostat, while pressure was controlled at 1 bar using the Berendsen barostat. Production run: A 100 ns MD simulation was run without restraints with the Berendsen thermostat and Parrinello‐Rahman barostat to ensure the adequate temporal resolution needed for tracking the dynamic protein–GO–HA interactions involved with ECM proteins and integrins. The Berendsen thermostat and barostat were used only during the equilibration phases to stabilise the temperature and pressure conditions, whereas the Parrinello–Rahman barostat was used during the production simulations. The extensive simulation framework examined various aspects of the GO–HA complex, including its structural stability, binding durability, and biomolecular flexibility in a biologically relevant context. The mechanistic insights into the osseointegration potential of GO‐HA coatings gained from this stage would help in their design and clinical application in dental implants. These simulations aimed to provide comparative insights into interaction stability and flexibility rather than predict any biological or clinical outcomes. The consistent treatment of material–biomolecule interfaces was ensured using GAFF2 for GO/HA and AMBER99SB for protein components within the same simulation framework.

### 2.6 Intermolecular Contact Analysis

To further examine the trends seen in the interaction of biomolecular partners with GO, HA and GO–HA, intermolecular atom‐pair contacts were analysed for the selected complexes. Contacts were grouped by atom type involved in the contact (CC, CO, CN, OO, NN, and NX where X represents contacts with the other heteroatom). These groups served as descriptors of hydrophobic, polar and hybrid hydrophobic/polar interactions between biomolecules and the surface. Counts for each complex were then compared to help describe the observed interaction trends. Tabulated data can be found in Supporting Information 2.

### 2.7 In Silico Toxicity Assessment of Graphene Oxide (GO) and Hydroxyapatite (HA)

In silico toxicity predictions were conducted for GO and HA as individual constituents, rather than for GO–HA as a combined material. The predictive toxicology assessment of individual constituents was performed using a comparative approach to elucidate the toxicological properties of GO and HA. This phase involved a comprehensive computational analysis of toxicological risks based on a wide range of molecular descriptors and parameters that characterise the structural, physicochemical, and electronic features. To screen for potential mutagenicity, tumorigenicity, irritation, and reproductive toxicity associated with GO and HA constituents, OSIRIS Data Warrior V6.4.2 [67] was employed, leveraging its advanced toxicity prediction models and seamless cheminformatics integration to predict toxicity. The software analyses functional groups and chemical substructures that result in adverse biological effects to facilitate a rudimentary safety assessment of nanocomposite building blocks. QikProp from the Schrödinger suite [68] was only used to generate selected physicochemical and ADMET‐related descriptor outputs of the modelled GO and HA structures, respectively. As OSIRIS DataWarrior and QikProp were primarily designed for small organic/drug‐like molecules, their use on model structures of graphene oxide and hydroxyapatite in the present study should be regarded with caution. These outputs are only used as preliminary qualitative assessments at the component level and not as definitive predictions of toxicity for the GO–HA nanocomposite. The correlation between toxicity and structural/pharmacophoric information provides a logical basis for rationally designing GO–HA coatings with minimised biological risks. These predictions were used qualitatively as preliminary indicators only and were not intended to reflect quantitative toxicological risk or validate nanomaterial safety assessment.

## 3 Results and Discussion

The interaction trends presented in this section originate from computational analyses and reflect molecular‐level patterns without predicting specific biological or clinical results.

### 3.1 Selection of Extracellular Matrix (ECM) and Integrin Receptors

The success of osseointegration critically depends on the establishment of stable interactions between the implant surface and ECM proteins and integrin receptors available at the bone–implant interface. The implant interface must support osteoblast adhesion and differentiation, as well as matrix deposition and mineralisation. For our molecular interaction studies, we considered a complete set of ECM proteins and integrin receptors involved in bone formation and implant integration. Table 1 shows the selected ECM proteins along with their functional binding residues, while Table 2 lists the integrin receptors included in our interaction analysis. This consistent selection allowed for a subsequent comparative analysis of protein–surface and integrin‐mediated interactions in the GO, HA, and GO–HA systems. ECM proteins and integrin receptors were then analysed to rationalise the interaction trends observed in the docking and molecular dynamics outcomes, as opposed to revalidating their biological rationale for selection. In contrast to previous studies that typically focus on protein adsorption or material properties in isolation, this study presents a computational framework that combines comparative docking, molecular dynamics simulations, interaction feature mapping, and in silico toxicity screening. The comparative docking‐based analysis reported in this study builds on the existing literature by focusing on how the integration of graphene oxide in hydroxyapatite affects the interaction trends across multiple ECM proteins rather than on individual protein–surface interactions.

**TABLE 1:** Extracellular matrix (ECM) proteins involved in osseointegration and their functional binding residues.

ECM protein	Function in osseointegration	PDB ID	Active site (residue number)
Biglycan	It regulates bone formation and remodelling and interacts with collagen and growth factors in the ECM	2FT3 [69]	91, 115, 120, 137, 144, 161, 170, 189, 215
Bone Sialoprotein	Promotes initial mineral deposition, binds αvβ3 integrin, and enhances osteoblast differentiation and matrix maturation	5CFA [70]	18, 21, 23, 25, 32
Collagen Type I	Primary organic component of bone ECM; provides structural scaffold for bone tissue; supports osteoblast adhesion via α2β1 integrin	7CWK [71]	11, 12, 13, 14
Decorin	Modulates collagen fibrillogenesis and indirectly supports matrix mineralisation and organisation	1XCD [72]	310, 311, 312, 315, 321, 324, 326
Fibronectin	It promotes osteoblast adhesion, spreading, and migration, binds integrins (especially α5β1), and enhances implant surface bioactivity	1FNF [73]	1363, 1364, 1365, 1388, 1389, 1391, 1418, 1419, 1501
Laminin	Supports cell adhesion via α6β1 integrin, promotes basement membrane stability, and influences early cell behaviour at the implant interface	4YEQ [74]	57, 84, 89, 90, 95, 96, 98, 115, 116, 118, 119, 167, 169, 170, 171
Osteocalcin	It regulates hydroxyapatite binding and mineralisation and is expressed by mature osteoblasts	1Q8H [75]	16, 19, 38, 39, 42, 43
Osteonectin	It binds calcium and hydroxyapatite, modulates collagen interactions, and enhances osteoblast differentiation and remodelling	1BMO [76]	65, 67, 77, 78, 79, 80, 81, 82, 83, 103, 107, 110, 111, 114, 115, 120
Osteopontin	It binds to αvβ3 integrin, regulates bone remodelling, mediates osteoclast adhesion, and is involved in mineralisation and cell recruitment	1MOY [77]	64, 65, 67, 68, 69
Periostin	It is involved in bone development and remodelling and modulates collagen cross‐linking and osteoblast differentiation	5YJG [78]	38, 39, 60, 64, 66, 67, 72, 78, 85, 110, 112, 123, 137, 138, 139, 142, 173, 195, 274, 318, 384, 410
Tenascin‐C	It is involved in tissue remodelling and modulates cell‐ECM interactions during early wound healing	6QNV [79]	1982, 1983, 2067, 2068, 2069, 2070, 2188, 2189, 2190
Vitronectin	It mediates cell adhesion and migration, binds integrins αvβ3 and αvβ5, and facilitates osteoblast attachment to implant surfaces	6O5E [80]	162, 163, 164, 207, 208, 209, 220, 227, 229, 239, 247, 255, 257, 347, 349

**TABLE 2:** Integrin receptors mediating cell–ECM interactions during osseointegration and their functional binding residues.

Integrin receptor	Ligand binding partner	Function in osseointegration	PDB ID	Active site (residue number)
α2β1	Collagen Type I	Major receptor for collagen; osteoblast attachment	1DZI [81]	153, 155, 221
α5β1	Fibronectin	Promotes osteoblast adhesion and spreading	3VI4 [82]	21, 23, 104, 168, 235, 291, 375, 438
α6β1	Laminin	Mediates binding of cells to the basement membrane	7CEC [83]	23, 97, 172, 240, 357, 417
αvβ3	Osteopontin, Vitronectin, Bone Sialoprotein	Cell adhesion, signalling, bone remodelling	1L5G [84]	95, 305, 323, 341, 367, 404, 579, 677
αvβ6	Fibronectin, LAP‐TGF‐β	Regulates inflammation and tissue remodelling	4UM8 [85]	167, 169, 170, 175, 179, 218, 219, 221
αvβ8	Osteopontin, Vitronectin	TGF‐β activation and matrix remodelling	6OM2 [86]	109, 123, 129, 144, 192, 241, 329, 332

The ECM proteins summarised in Table 1 belong to a complex family of structural and functional molecules that modulate cellular events during osseointegration. Biglycan and decorin are members of the small leucine‐rich proteoglycan (SLRP) family, which are involved in collagen fibrillogenesis and regulation of growth factor activity [87, 88]. These ECM proteins regulate osteoblast activity and matrix organisation. Thus, they are among the most suitable candidates for monitoring the bioactivity of surfaces [89]. Bone sialoprotein (BSP) and osteopontin are non‐collagenous RGD‐rich proteins that mediate osteoblast and osteoclast adhesion through integrin recognition, specifically αv integrin‐containing subtypes [90, 91]. Fibronectin, an RGD‐containing glycoprotein, is known to bind to a large number of integrins. It is also essential for early cell attachment, migration, and matrix organisation during bone repair [92].

Collagen Type I is the most abundant organic constituent of the bone ECM, which, in addition to offering mechanical integrity to the tissue, also provides biochemical signals for cellular attachment. The collagen Type I matrix primarily interacts with integrin α2β1, representing a suitable scaffold for matrix deposition [93, 94]. Laminin is an ECM protein of the basement membrane that was also considered for its role in osteoblast adhesion through binding to integrin α6β1 [95]. Proteins such as osteocalcin and osteonectin (SPARC) regulate calcium homeostasis and hydroxyapatite deposition, contributing to mineralisation on implant surfaces [96]. In contrast, periostin and tenascin‐C are ECM proteins that are highly upregulated during bone repair and remodelling and can influence the expression of osteogenic markers and cell‐matrix interactions [97, 98]. Finally, vitronectin is a multifunctional adhesive glycoprotein that can mediate osteoblast attachment through integrin‐mediated mechanisms and increase the surface bioactivity of implant materials [99].

On the receptor end, as shown in Table 2, we selected integrins known to be upregulated in osteoblasts or other bone‐resident cells, as well as integrins that have been reported to interact with the selected ECM proteins. Integrin α2β1, for instance, is known to bind collagen Type I and mediate the adhesion and spreading of osteoblasts on biomaterial surfaces [100]. Integrin α5β1, the primary receptor for fibronectin, supports osteoblast differentiation and ECM organisation [101]. Integrin α6β1, which binds to laminin, plays a role in early stage cell adhesion [102]. Simultaneously, αvβ3 and αvβ5 are recognised for their ability to bind RGD‐containing ligands, such as osteopontin, BSP, and vitronectin, which are involved in promoting osteogenic signalling [103, 104]. Additional integrins included in Table 2, such as αvβ6 and αvβ8, contribute to ECM remodelling and the activation of transforming growth factor‐beta (TGF‐β), further highlighting their relevance in modulating bone repair and regeneration [105]. The integrins listed above were selected based on their known amino acid residues involved in ECM ligand recognition, and these residues are presented here to provide a biological context for interpreting the docking results rather than as predefined docking constraints. Overall, this study provides a solid basis for assessing how different dental or orthopaedic biomaterials interact with the bone microenvironment using a more meticulous set of biologically relevant ECM‐integrin pairs.

### 3.2 3D Structure Modelling of Graphene Oxide (GO) and Hydroxyapatite (HA) With MM2 Energy Minimisation

The structures were minimised using the MM2 force field to describe the molecular geometry and structural stability of GO and HA. The classical molecular mechanics approach, which optimises molecular features such as bond lengths, bond angles, torsion angles, and nonbonded interactions between molecules, leads to the lowest energy conformers that are more likely to be biologically relevant. The energetic parameters listed in Table 3 provide information on the mechanical and electronic stabilities of both nanomaterials. Integrin receptor profiles were used to provide context and assist in the interpretation of the trends of protein–surface interactions in the docking and molecular dynamics studies described below.

**TABLE 3:** Comparison of energy parameters between Graphene Oxide (GO) and hydroxyapatite (HA) following MM2 energy minimisation.

Parameter (kcal/mol)	Graphene oxide (GO)	Hydroxyapatite (HA)
Stretch	9.4512	0.1084
Bend	35.8118	0.2093
Stretch‐Bend	0.6507	0.0017
Torsion	39.0070	2.3385
Non‐1,4 VDW	−8.1136	−0.2020
1,4 VDW	17.0332	−0.3516
Dipole/Dipole	−4.5438	−12.6958
Total Energy	89.2964	−10.5914

GO displayed a very high total energy (89.2964 kcal/mol), whereas HA had a negative total energy (−10.5914 kcal/mol). These findings indicate that the materials have different molecular packing and electronic interactions. The positive total energy value of GO indicates a strained structure with a high energy density owing to the large π‐electron framework and oxygen functional groups such as epoxide, hydroxyl, and carboxyl groups, while a planar hexagonal carbon framework restricts the conformational freedom. The stretch, bend, and torsional energies of GO were significantly higher than those of HA, with values of 9.4512, 35.8118, and 39.0070 kcal/mol, respectively, compared to 0.1084, 0.2093, and 2.3385 kcal/mol for HA, respectively. This indicates that GO is under a lot of internal stress in its carbon skeleton as well as functional groups. The high torsional energy of the GO structure indicates that it has a low rotation capability. This also results in steric rigidity, which can influence its ability to adapt to the protein surface upon contact. The 2D nature of GO facilitates surface anchoring. The GO molecule had a high 1,4 VDW energy of 17.0332 kcal/mol, indicating strong steric interactions between atoms that are three bonds away. The combination of the condensed structural arrangement and the location of the oxygen functional groups results in an elevated total energetic tension. HA, a naturally occurring calcium phosphate, exhibited negligible stretching, bending, and torsion energy contributions. The material has an energetically favourable structure consistent with its crystalline arrangement and ionic bonding network, leading to structural stability with minimal internal strain. HA showed favourable nonbonded interactions, including non‐1,4 van der Waals and dipole–dipole interactions. The non‐1,4 VDW energies of GO and HA were −8.1136 and −0.2020 kcal/mol, respectively, and the dipole–dipole interaction energies were −4.5438 and −12.6958 kcal/mol, respectively. The higher polarity of HA is due to its more negative dipole interaction resulting from calcium and phosphate ions, which give rise to electrostatic interactions with proteins and cell membranes that are needed for osseointegration.

### 3.3 Results of the Molecular Docking Simulation

**FIGURE 1:**
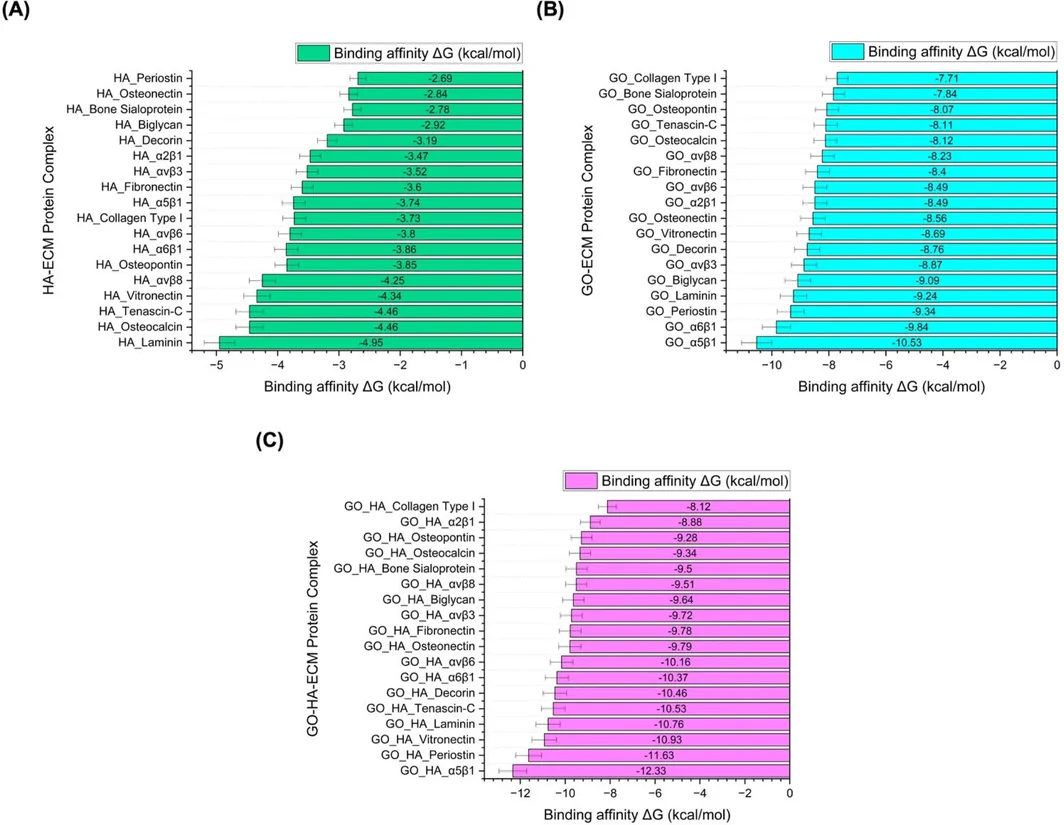
Molecular docking simulation results showing the predicted binding affinity (ΔG, kcal/mol) of extracellular matrix (ECM) proteins interacting with various nanocomposite materials. (A) HA–ECM complex. (B) GO–ECM complexes. (C) GO–HA–ECM.

We performed molecular docking simulations to gain insights into the binding modes of HA and GO to various ECM proteins and integrins. The docking simulations showed significant differences in the binding affinities, interaction energies, and conformational stability according to the type of biomolecule. We evaluated the molecular interactions in terms of HADDOCK scores and binding affinity (ΔG) with cluster size and RMSD values, as well as contributions from van der Waals, electrostatic, desolvation, and buried surface area energies. All docking simulation results are presented in Supporting Information 1 (Figure 1).

HA_Laminin was the best binder among the HA‐based complexes, both in terms of the HADDOCK score (−19.7) and binding affinity (−4.95 kcal/mol). The second and third best‐scoring HA‐based complexes were HA_Collagen Type I (HADDOCK score: −18.2; binding affinity (ΔG) −3.73 kcal/mol) and HA_Tenascin‐C (HADDOCK score: −16.4; binding affinity (ΔG) −4.46 kcal/mol). The best binding of HA_Laminin can be explained by its large electrostatic contribution (−186.5 kcal/mol) and favourable desolvation energy (6.2 kcal/mol). The binding of HA to various ECM proteins involves the formation of electrostatic and H‐bonding interactions with polar residues in a stable manner. The two integrin‐binding partners, HA_αvβ8 and HA_α6β1, revealed moderate binding scores of −15.0 and −12.4, respectively. However, the integrins buried larger surface areas and had smaller RMSD values, suggesting a more compact docking conformation. Among the integrin complexes, HA_α5β1 showed the poorest HADDOCK score (−11.6) and a relatively smaller van der Waals energy contribution of −1.4 kcal/mol, which may indicate that its surface of interaction with HA is unfavourable.

**FIGURE 2:**
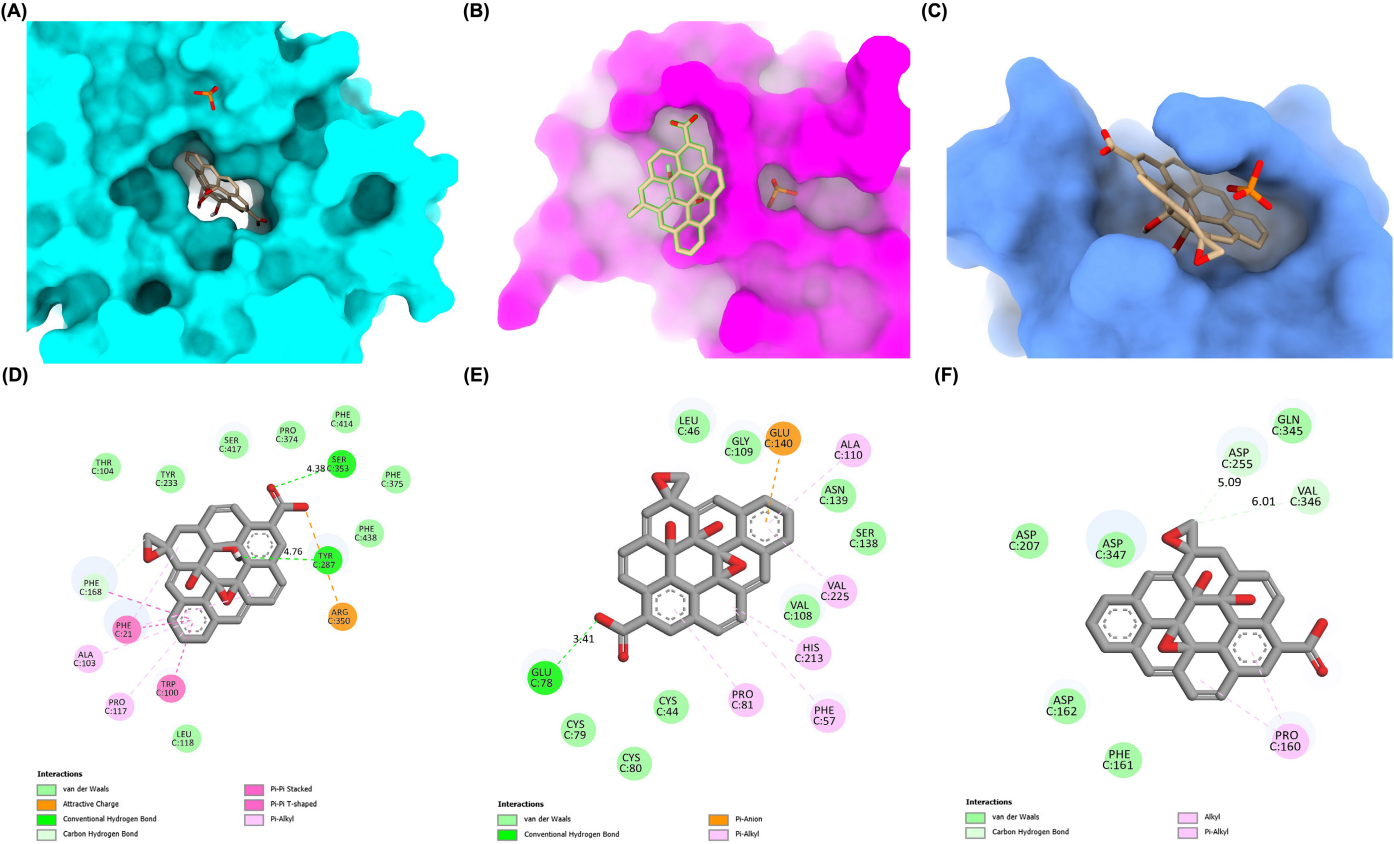
Molecular docking representations of selected biomolecule–GO–HA surface complexes. (A) 3D perspective of the GO_HA_α5β1 complex. (B) 3D perspective of the GO–GO‐HA–periostin complex. (C) 3D perspective of the GO_HA‐vitronectin complex. (D) 2D interaction map of the GO_HA_α5β1 complex. (E) 2D interaction map of the GO–GO‐HA–periostin complex. (F) 2D interaction map of the GO_HA_Vitronectin complex. The 3D panels illustrate the predicted biomolecule‐facing association with the combined GO–HA surface representation, whereas the two‐dimensional maps highlight individual residue‐level interactions identified in the docking poses.

The GO‐derived complexes showed a significant increase in binding affinity and docking scores (DS), with improved potential for better interaction with HA. The GO_α5β1 docking profile showed the most favourable outcome, with a HADDOCK score of −56.0 and an extremely high binding affinity (BA) of −10.53 kcal/mol. The strong interaction reached a high magnitude because of the summative effect of the high van der Waals energy (−33.7 kcal/mol) and large electrostatic energy (−53.3 kcal/mol), which established potent physical and electrostatic forces. The complex exhibited the largest buried surface area of 745.4 Å^2^ compared to the other complexes, indicating a larger contact interface with the potential for increased biological adhesion. GO_α6β1 and GO_Laminin showed high binding profiles with HADDOCK scores of −42.1 and −43.6, respectively, and ΔG values of −9.84 and −9.24 kcal/mol, respectively. GO_α6β1, similar to GO_α5β1, also revealed the second largest buried surface area with a value of 617.6 Å^2^ and a significant electrostatic interaction energy of −66.2 kcal/mol, indicating a stable and highly interactive structure. The interaction between GO and periostin was favourable, but it showed a higher range of variation in DS values (higher SD) and also represented a strong interaction. Collectively, these results demonstrate that graphene oxide has a stronger interaction potential with essential ECM proteins and integrins than hydroxyapatite. The functional surface chemistry of GO and its larger available surface area enhance the van der Waals and electrostatic interactions (Figure 2).

The predicted interactions of the combined GO–HA surface representation with ECM proteins and integrin receptors showed more favourable docking profiles than the corresponding GO‐ or HA‐only systems. The GO_HA_α5β1 complex showed the highest docking score, with a HADDOCK score of −71.4 ± 2.2 and a favourable binding free energy (ΔG) of −12.33 kcal/mol, suggesting a strong thermodynamic association. The complexes with the highest van der Waals and electrostatic contributions and the maximum buried surface area (915.3 ± 12.2 Å^2^) compared to all other complexes indicated close contact between molecules in the complex and high stability. A hydrogen bond was formed between the GO hydroxyl group and Tyr287 residue, which was 4.76 Å away (Figure 2D). The complex is further stabilised by other attractive charge interactions between the main hydroxyl group and Arg350, van der Waals interactions, and π–π stacking. Incorporating GO into HA matrices significantly improved the binding performance over previously characterised HA‐ECM and GO‐ECM complexes. The GO‐HA hybrid exhibited synergistic properties, resulting in increased binding affinities for ECM proteins, such as periostin, fibronectin, and laminin, along with integrins α5β1, αvβ3, and αvβ6. The GO_HA_ periostin and GO _ HA‐vitronectin complexes had high binding affinities, as suggested by ΔG of −11.63 and −10.93 kcal/mol, respectively. The HA‐only complexes had weaker binding ranging between −8 and −9 kcal/mol but showed notable improvement when compared with the GO_HA complexes. GO_HA complexes have more favourable electrostatic energy interactions, which showed particular improvements in the α5β1 and periostin interfaces from the surface charge and functional groups of GO, such as carboxyl, hydroxyl, and epoxide groups, which further enhance polar interactions and hydrogen bonding with target proteins. The GO_HA_Periostin complex has a hydrogen bond between the hydroxyl group of GO and Glu78, which is 3.41 Å apart (Figure 2B,E). The GO hydroxyl group formed carbon–hydrogen bonds with Asp255 and Val346 residues in the GO_HA_Vitronectin complex, which are 5.09 and 6.01 Å away, respectively (Figure 2C,F). The observations shown in Figure 2C,F indicate extra stabilisation from weak polar interactions. The GO_HA_Tenascin‐C and GO_HA_Decorin complexes showed ΔG values of −10.53 and −10.46 kcal/mol, respectively, which is an improvement over the ΔG values of GO and HA used individually. The docking poses also showed conformational stability, which can be understood by their large buried surface areas and low RMSD values. The GO‐HA conjugate improved the binding strength and stabilised protein interactions at the bio‐interface. The GO_HA_α6β1 and GO_HA_αvβ6 complexes have high affinity, as suggested by Δ*G* values of −10.37 and −10.16 kcal/mol, respectively, which outperform the HA‐only complexes, which show Δ*G* values of less than −9.0 kcal/mol. Through mechanical reinforcement and chemical bioactivity, which act in concert, the GO‐HA combination enhances cell adhesion to ECM scaffolds mediated by proteins, which is essential for dental tissue engineering applications, including bone regeneration and implant integration.

The interactions between GO‐HA complexes and ECM proteins and integrin receptors were further analysed to understand the detailed contacts across different atom pairs. A comparison was made among atom pair types, such as CC, CO, and CN. CC and CO contacts were found to be the most significant among all atom pair contacts of HA‐ECM or HA‐integrin interactions, which depicted the prevalence of hydrophobic and polar contacts, respectively. A total contact of 3209 CC and 1418 CO pairs was noted for the GO_HA_α5β1 system with the highest interaction number, suggesting a large interaction surface area or more appropriate conformational fit. Integrin complexes GO_HA_αvβ3 and GO_HA_αvβ6 exhibited high contact numbers for CC, CO, CN, and OO interactions, which further supports the strong binding interactions observed for these complexes. The GO_HA_Collagen Type I and GO_HA_α6β1 systems recorded low interaction numbers for CN and OO, most likely because of the incompatibility in electrostatics or hindered access of the side chains. We found near‐zero NN and NX interactions for all systems, which are not preferred because of steric and energy considerations. A detailed tabulation of all the intermolecular contacts is provided in Supporting Information 2.

### 3.4 3D Pharmacophore Modelling to Assess Active Functional Groups of Graphene Oxide (GO) and Hydroxyapatite (HA)

**FIGURE 3:**
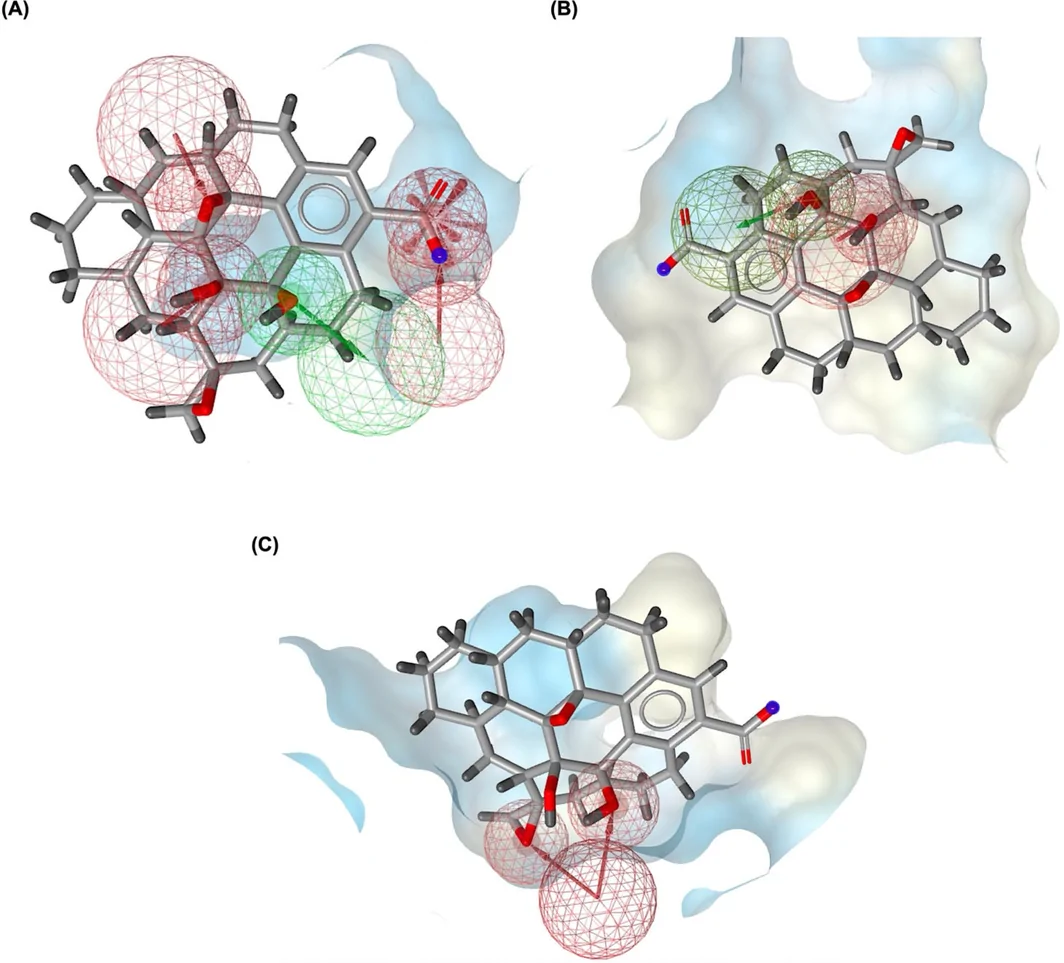
Pharmacophore interaction‐feature maps derived from selected biomolecule–GO–HA docking complexes. (A) 3D pharmacophore model of the GO_α5β1complex. (B) 3D pharmacophore model of GO‐HA–periostin complex. (C) 3D pharmacophore model of the GO_HA‐vitronectin complex. Hydrophobic interactions are represented by yellow spheres, hydrogen bond donors by green arrows, and hydrogen bond acceptors by red arrows (with spheres), illustrating the key molecular interaction features of each complex. These maps are intended to characterise biomolecule–surface recognition and do not represent direct structural visualisation or quantitative measurement of the GO–HA interfacial bond.

This study investigated the molecular association between GO and HA with ECM proteins. The 3D pharmacophore models of three major GO‐HA‐ECM complexes, GO_HA_α5β1, GO_HA_Periostin, and GO_HA_Vitronectin, were built based on the optimised docking conformations and defined with important geometric and chemical interaction features that influence molecular recognition. The key interactive regions of GO and HA involved in protein affinity were discerned by extensive mapping of important pharmacophoric features (hydrophobic contacts, hydrogen bond donors, and acceptors of each complex), as shown in Figure 3. The pharmacophore model of the GO _α5β1 complex in Figure 3A features the presence of hydrogen‐bond acceptors (red spheres and mesh) around the epoxide and hydroxyl functional groups of GO. A dense web of hydrogen bond donors (green mesh), presumably contributed by HA moieties, was also visible. Hydrophobicity (yellow), which is not depicted here, is often observed in close proximity to aromatic systems. It is typically associated with π–π or van der Waals interactions with the hydrophobic residues of integrin and is present in close proximity to the GO aromatic backbone.

The GO‐HA periostin complex (Figure 3B) generated a pharmacophore that showed an even distribution of hydrogen bond acceptors and donors, resulting in a stable interface. The 3D arrangement of pharmacophoric features suggested that periostin established strong adhesive interactions with GO and HA via a synergistic hydrogen‐bond network to form an effective anchor. The acceptor groups were distributed as clusters on the aspartate/glutamate residues of periostin, and the donor groups were lining the phosphate groups of HA, thus suggesting the importance of the surface chemistry of GO for molecular recognition. The GO_HA_Vitronectin complex (Figure 3C) had a higher abundance of hydrogen bond acceptors in the periphery of the oxygen functionalities of the GO sheet. The intermolecular interactions appeared to intensify the affinity for the polar residues of vitronectin. The β‐sheet regions of the protein aligned closely with extended hydrophobic regions, facilitating structural compatibility through nonpolar interactions to anchor the protein to the GO‐HA hybrid.

Hydrogen bonding and hydrophobic interactions were identified as significant forces for GO‐HA binding to ECM proteins in 3D pharmacophore models. The presence of hydrogen bond acceptors in all the complexes shows the importance of GO's oxygen‐rich functionalities, while HA provides stabilizing donor sites created by calcium‐bound hydroxyl or phosphate groups. This study illustrates how the chemical synergy between GO and HA creates a reactive surface that strongly interacts with ECM proteins. This is significant for dental applications because robust integration into the extracellular matrix promotes cell adhesion, proliferation, and differentiation to facilitate periodontal regeneration, implant osseointegration, and dentine‐pulp complex regeneration. The enhanced binding profiles in the pharmacophore models show that GO‐HA composites can act as a multipurpose scaffold for dental tissue engineering to enhance the performance of biomaterials.

### 3.5 Results of the Molecular Dynamics (MD) Simulation

**FIGURE 4:**
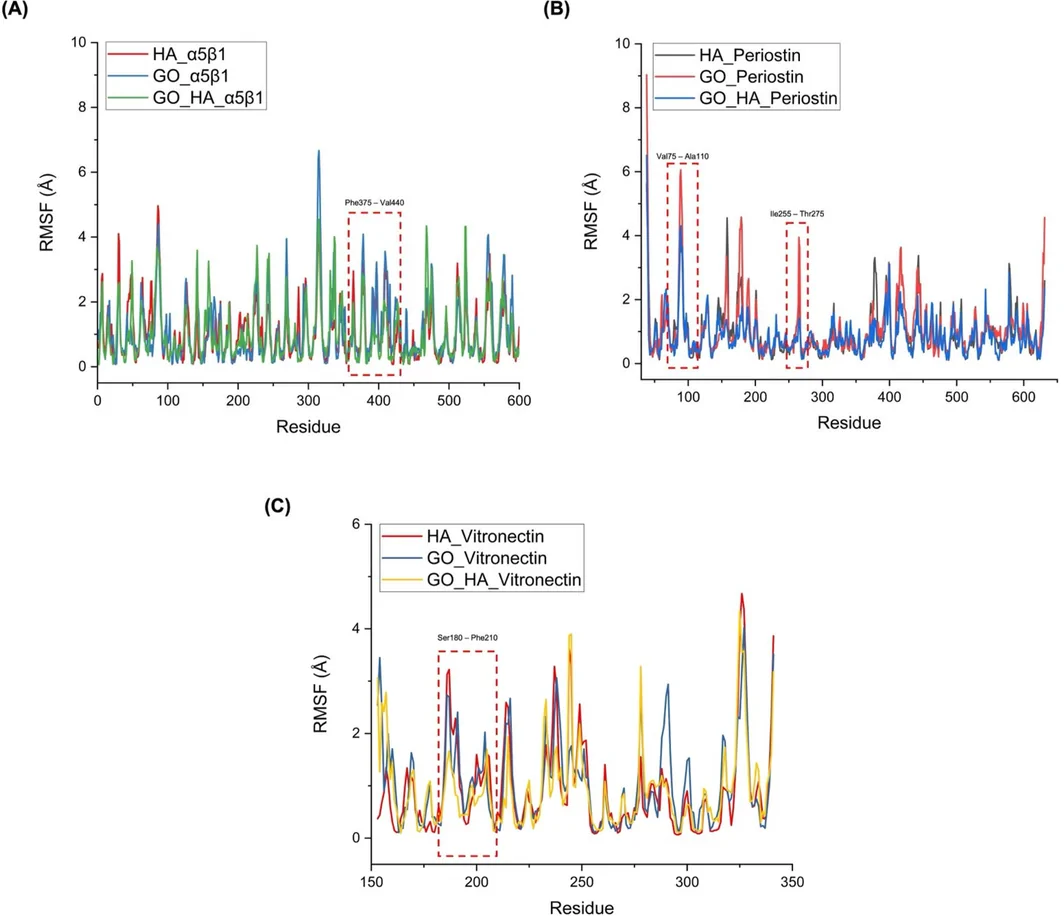
Molecular dynamics (MD) simulation. (A) RMSF profiles of the α5β1 complexes. (B) RMSF profile of periostin. (C) RMSF profiles of the vitronectin complexes.

MD simulations were conducted to explore the structural stability of the three major ECM protein complexes (α5β1 integrin, Periostin, and Vitronectin) with HA, GO, and their combination (GO_HA). The root‐mean‐square fluctuation profiles of all the systems are shown in Figure 4. Table 4 summarises the time‐averaged parameters, including the RMSD, RMSF, radius of gyration (RoG), and hydrogen bond numbers. The backbone fluctuations in all systems were uniform through the RMSF profiles of the α5β1 integrin complexes, whereas residues 375–440 showed high peaks (red box, Figure 4A). The GO_HA_α5β1 complex showed reduced fluctuations compared to both HA and GO systems, which may be a result of improved local stabilisation due to an increase in protein‐nanomaterial interactions. The GO_HA_α5β1 complex had the highest average RMSD value of 1.584 nm. This indicates an increase in global structural rearrangement over time. This increase is not deleterious because the lower RMSF value of 1.019 nm indicates stabilisation at the level of individual residues. The 1.735 nm RoG value suggests a relatively more extended conformation of the complex, which may reflect adaptation to a larger surface contact area, supporting the discovery that this nanomaterial combination created stronger and more stable interactions.

**TABLE 4:** Time‐averaged structural properties obtained from molecular dynamics (MD) simulations of the top three GO‐HA‐ECM complexes.

Complex	Average RMSD (nm)	Average RMSF (nm)	Average RoG (nm)	Number of hydrogen bonds
HA_α5β1	1.021	1.033	1.472	12
GO_α5β1	1.231	1.102	1.608	15
GO_HA_α5β1	1.584	1.019	1.735	22
HA_Periostin	1.105	0.973	1.224	10
GO_Periostin	1.142	1.041	1.709	17
GO_HA_Periostin	1.313	0.852	1.849	20
HA_Vitronectin	1.147	0.926	1.310	11
GO_Vitronectin	1.349	1.024	1.635	14
GO_HA_Vitronectin	1.478	0.924	1.911	19

The Periostin protein has distinct flexible regions between the Val75, Ala110, and Ile255‐Thr275 residue sequences (Figure 4B). The local structural stability of periostin increased when it was complexed with the GO_HA complex, as it had lower RMSF values than when complexed with HA or GO alone. The dual‐nanomaterial system created additional contact points, resulting in the observed loss of flexibility. The GO_HA_Periostin complex had the lowest RMSF (0.852 nm) among all periostin systems, despite a moderate increase in RMSD (1.313 nm), suggesting increased stability at the residue level. The structure had the largest RoG value of 1.849 nm, implying that it retained an expanded but stable form. The GO_HA combination had a stronger interaction capacity, forming 20 hydrogen bonds, which were double those of HA_Periostin.

RMSF analysis of vitronectin demonstrated that the GO_HA_Vitronectin complex better stabilised the Ser180‐Phe210 region of vitronectin than either GO or HA (Figure 4C). The stabilisation of such regions is important because it retains the functional domains of proteins. GO‐HA‐vitronectin displayed the highest RMSD value of 1.478 nm, indicating enhanced conformational flexibility. However, this complex exhibited the lowest RMSF (0.924 nm) and the highest number of hydrogen bonds (19), confirming that the ternary nanomaterial complex resulted in substantial stabilisation of the protein. The system demonstrated the highest RoG value at 1.911 nm, conforming to the trend observed for the other two systems. This was due to alterations that accommodated GO_HA binding. Overall, MD simulations showed that the co‐application of GO and HA created a more favourable environment for ECM proteins than the application of the separate components. The GO_HA complexes showed modest increases in RMSD and RoG values, indicating structural modifications with controlled adaptation. The reduced RMSF and increased number of hydrogen bonds reflected enhanced local residue stability and stronger molecular interactions.

### 3.6 In Silico Toxicity Assessment of Graphene Oxide (GO) and Hydroxyapatite (HA)

In silico studies of GO and HA showed significant differences between the two materials in their physicochemical and toxicological properties (Table 5). The calculated cLogP of GO was 2.330, indicating intermediate lipophilicity and the potential to cross biological barriers. In contrast, HA showed strong hydrophilicity with a cLogP of −8.782, in line with its inorganic, ion‐based structure. Consistent with this, the solubility results showed a separation between the GO and HA components. GO is relatively insoluble (cLogS = −5.375), whereas HA has strong water solubility (cLogS = 1.958). This favours the bioavailability and distribution in biological systems. The calculated total surface area of GO was 290.060 Å^2^, which was significantly higher than that of HA (59.860 Å^2^). The polar surface area (PSA) of GO was 102.820 Å^2^. This suggests a high density of functional groups on the GO surface. This provides a molecular basis for the high functionalisation capacity of GO. The properties of GO that contribute to its reactivity also indicate high in silico toxicity. GO is predicted to be a hazard with regard to mutagenicity, tumorigenicity, effects on reproduction, and irritation. In contrast, HA has no predicted toxicological endpoints. It is worth noting that toxicology studies have been conducted on GO and HA independently. This is a limitation of in silico approaches, as the current platforms do not have a means to model the GO–HA composite directly. Thus, the prediction of reduced toxicity is indirect and requires experimental validation. This raises important safety considerations for the use of GO in biomedical applications, such as dentistry and regenerative medicine. Biocompatibility is the most critical requirement for these applications.

**TABLE 5:** In silico toxicity results for (GO) and hydroxyapatite (HA).

Parameter	Graphene oxide (GO)	Hydroxyapatite (HA)
cLogP	2.330	−8.782
cLogS	−5.375	1.958
Total surface area (Å^2^)	290.060	59.860
Polar surface area (Å^2^)	102.820	96.060
Relative PSA	0.302	1.000
Mutagenic	High	None
Tumorigenic	High	None
Reproductive effect	High	None
Irritant	High	None
Shape index	0.342	0.600
Molecular flexibility	0.215	0.100
Molecular complexity	1.101	0.346
Solvent accessible surface area (SASA) (Å^2^)	755.812	132.457
Hydrophobic component of SASA (FOSA) (Å^2^)	643.158	85.326
Hydrophilic component of SASA (FISA) (Å^2^)	112.654	47.131
QPlogHERG	−4.517	−1.238
QPPCaco	145.392	28.743
QPlogBB	−2.486	−1.804
QPPMDCK	78.216	20.187
QPlogKp	−2.103	−3.528
QPlogKhsa	−1.117	−2.014

A detailed structural analysis of GO revealed its higher molecular complexity (1.101) and surface accessibility (SASA = 755.812 Å^2^), with the most hydrophobic surface area (FOSA = 643.158 Å^2^). The physicochemical properties of GO may contribute to its harmful interactions with the cellular membrane and DNA, thus attributing to its potential genotoxic and carcinogenic effects. The QPlogHERG value of −4.517 for GO may be attributed to its moderate cardiotoxicity risk due to HERG channel inhibition, which is a standard test during safety evaluations for any drug. The moderate permeability of GO across different cellular barriers in the experimental findings (QPPCaco = 145.392; QPPMDCK = 78.216) in tandem with its very low penetration through the blood–brain barrier (QPlogBB = −2.486) might indicate that the effects of GO are likely to be localised in the body when used as scaffolds or coatings. The HA data showed safety for all analysed parameters. The substance showed low molecular flexibility and complexity in tandem with a low permeability (QPPCaco = 28.743) and a very low risk for HERG channel inhibition. The actual use of the material as a bone graft material and dental restorative material in a clinical setup is further supported by its nonmutagenic and nonirritant properties. Although the findings of the current study indicate that the incorporation of GO into the HA matrix may help in passivating the reactive surface groups, thus improving biocompatibility, this is just a theoretical postulate, and the actual safety of the GO–HA composite still needs to be proven through experimental approaches such as cytotoxicity assays and in vivo analyses.

The cytotoxicity of GO has been largely ascribed to various factors, such as nanosheet size, surface charge, level of oxidation, and dose‐dependent responses. Experimental evidence has demonstrated that smaller GO flakes (< 200 nm) are internalised more easily by cells, leading to mitochondrial dysfunction, oxidative stress, and membrane damage [106, 107]. Moreover, the abundance of oxygen‐containing functional groups (hydroxyl, epoxide, and carboxyl groups) on GO surfaces results in a highly negative charge, which promotes the production of reactive oxygen species (ROS) and inflammation. These combined properties have been linked to the mutagenic, tumorigenic, and irritant effects of GO, as observed in some studies. In contrast, HA provides countermeasures to GO's cytotoxicity because of its intrinsic biocompatibility and bioactivity. The calcium phosphate surface of HA neutralises the excessive negative charge of GO, masking its highly reactive oxygen functionalities, thereby limiting ROS production. Furthermore, HA creates a stable osteoconductive microenvironment that promotes cell adhesion and proliferation, helping to mitigate the cytotoxic effects of GO. Therefore, GO was embedded into HA composites to enhance its bioactivity and biocompatibility.

## 4 Limitations and Future Works

The results of this study must be contextualised within the limitations inherent to in silico research methodologies. While the current investigation sheds light on the intricate molecular dynamics that may dictate the interactions between GO and HA coatings and ECM proteins, it is essential to note that such computational approaches, including molecular docking, pharmacophore modelling, and MD simulations, are confined to theoretical speculations at the molecular and atomic levels. The dynamic and multifactorial nature of in vivo osseointegration, encompassing not only the biochemical environment but also biomechanical forces and host immune responses, is not captured by these silico models. Factors such as competitive adsorption from serum proteins, cellular immune reactions, temporal ECM remodelling, and mechanical loading on dental implants remain unaddressed in the computational framework. In addition, the present study does not include experimental characterisation or biological validation of GO–HA coatings. Specifically, material‐level properties and coating morphology, composition, crystallinity, and surface chemistry were not experimentally evaluated using techniques such as scanning electron microscopy (SEM), transmission electron microscopy (TEM), X‐ray photoelectron spectroscopy (XPS), or X‐ray diffraction (XRD). A further limitation concerns the structural representation of the GO–HA nanocomposite. The present computational model does not establish the precise nanoscale architecture, covalent bonding, or intrinsic GO–HA interfacial stabilisation of a fabricated coating. Rather, GO and HA were represented as component‐level molecular structures within a simplified interfacial model designed to investigate comparative biomolecule–surface interactions. Consequently, the present docking, pharmacophore, and molecular dynamics calculations should not be interpreted as direct structural validation of a physically fabricated GO–HA nanocomposite or as quantitative evidence of the intrinsic cohesion of the GO–HA interface. Experimental characterisation will be necessary to determine the actual morphology, chemical composition, surface chemistry, crystallinity, and interfacial organisation of fabricated GO–HA coatings.

Consequently, while the current study advances our understanding and presents a compelling narrative on potential interaction patterns at the nano‐bio interface, it should not be misconstrued as a conclusive determination of the biological efficacy. The necessity for experimental validation is paramount and will be the focus of subsequent research. Experimental studies will be required to determine whether the molecular interaction patterns predicted in the present work translate into measurable changes in material properties, protein adsorption, cellular responses, and osseointegration. At the in vitro level, assays for protein adsorption and adhesion will be pivotal in experimentally validating whether periostin, fibronectin, vitronectin, and integrins exhibit enhanced binding to GO–HA compared to HA. Surface characterisation techniques, including atomic force microscopy, scanning electron microscopy, and X‐ray photoelectron spectroscopy, can provide empirical data to correlate the predicted molecular interactions with the surface topography and chemistry of the nanocomposite coatings. Cell culture experiments with osteoblast‐like cells will provide biological insights into the predicted adhesion mechanisms mediated by integrin binding, particularly through cell adhesion assays and evaluations of osteogenic differentiation. Regarding osteoblast viability, standard cytotoxicity assays, such as MTT, CCK‐8, and Live/Dead staining, will provide both quantitative and qualitative assessments of cell viability in the presence of GO–HA coatings, thus substantiating the cytocompatibility predictions. Additionally, the assessment of macrophage responses and cytokine profiles will be an integral part of the experimental validation to ensure that the osteoconductive properties of the GO–HA coatings are not overshadowed by adverse immunological reactions.

In vivo studies will be the next step in the validation process, and animal implantation models will be crucial in evaluating the osseointegration performance of GO–HA coatings under physiological conditions. Quantitative assessments using techniques such as micro‐computed tomography (micro‐CT), histomorphometric analysis, and biomechanical pull‐out tests provide data on bone–implant contact, new bone formation, and mechanical stability. Additionally, in vivo studies should consider systemic interactions, such as the biodistribution of graphene oxide particles, durability of the coating under cyclic mechanical loading, and possible antibacterial effects in the context of peri‐implant infections. In the long term, the translation of GO–HA nanocomposite coatings to the clinic will require the assessment of biological efficacy, reproducibility of coating fabrication processes, and scalability to real implant geometries. The optimisation of the coating thickness, flake size, and oxidation state of graphene oxide is necessary to maximise bioactivity while minimizing cytotoxicity. Long‐term studies are needed to evaluate the durability, immunological safety, and osseointegration of implants under dynamic conditions that mimic the oral environment. These experimental and translational studies will be necessary to determine whether the molecular‐level trends identified computationally in the present work can be reproduced at the material, cellular, and organismal levels. Therefore, the current findings should be regarded as a computational framework for generating testable hypotheses rather than as evidence of established biological or clinical efficacy. By integrating computational predictions with systematic experimental validation, future studies can bridge the gap between in silico findings and the translational evaluation of GO–HA coatings for dental implant applications.

## 5 Conclusion

In this study, molecular docking, molecular dynamics simulation, interaction feature mapping, and in silico toxicity screening were integrated to form a virtual assessment of GO–HA coatings from the nanoscale perspective, with special interest in surface–biomolecule interactions for osseointegration. Docking analysis revealed that the interaction trends were more promising for GO–HA for the majority of extracellular matrix proteins in the screened panel. In addition, the docking results were further validated by MD simulation in terms of the enhanced stability and persistence of the surface–protein interaction during the simulated period. The mapping of interaction features based on protein pharmacophores provided qualitative information on the role of surface chemical functionalities in the protein interaction pattern, and component‐level preliminary trends for GO–HA were suggested by in silico toxicity screening. The proposed integrated in silico approach could be a good complement to experimental studies for assessing material surface modifications.

## Author Contributions

**Rayan Ibrahim H. Binduhayyim:** data curation, formal analysis, writing – original draft, visualization. **Masroor Ahmed Kanji:** conceptualization, methodology, writing – original draft, funding acquisition. **Artak Heboyan:** validation, formal analysis, writing – review and editing, project administration, supervision. **Ravinder S. Saini:** conceptualization, methodology, investigation, resources, writing – review and editing, supervision, project administration. **Doni Dermawan:** conceptualization, methodology, validation, formal analysis, investigation, resources, writing – review and editing, software. **Syed Altafuddin Quadri:** data curation, formal analysis, writing – original draft.

## Funding

The authors extend their appreciation to the Deanship of Research and Graduate Studies at King Khalid University for funding this work through Large Research Project under grant number RGP2/405/47.

## Ethics Statement

The authors have nothing to report.

## Consent

The authors have nothing to report.

## Conflicts of Interest

The authors declare no conflicts of interest.

## Supporting information


**Supporting Information: 1** Molecular docking results.

**Supporting Information: 2** Molecular interactions.

## Data Availability

The data supporting the findings of this study are available from the corresponding author upon request.
